# Unilateral pulmonary vein atresia presenting with recurrent haemoptysis in a child: a case report

**DOI:** 10.1186/s12887-020-02348-7

**Published:** 2020-09-24

**Authors:** Martin Ngie Liong Wong, Ing Ping Tang, Yek Kee Chor, Kiew Siong Lau, Anne Rachel John, King Ching Hii, Olive Pei Yi Lee, Wooi Kok Lim, Hannah Pei Koon Tan

**Affiliations:** 1 Paediatric Cardiology Unit, Sarawak Heart Center, Kota Samarahan, Malaysia; 2 Department of ORL HNS, Faculty of Medicine and Health Sciences, Universiti Malaysia Sarawak, Kota Samarahan, Sarawak Malaysia; 3 Department of Paediatric, Sarawak General Hospital, Kuching, Malaysia; 4 Department of Radiology, Sarawak General Hospital, Kuching, Malaysia; 5 Department of Paediatric Surgery, Sarawak General Hospital, Kuching, Sarawak Malaysia; 6 Department of Paediatric, Miri Hospital, Miri, Malaysia

**Keywords:** Haemoptysis, Pulmonary vein atresia, Bronchial varices

## Abstract

**Background:**

Haemoptysis is an uncommon presenting symptom in children and is usually caused by acute lower respiratory tract infection or foreign body aspiration. We report a rare case of right unilateral pulmonary vein atresia (PVA) as the underlying aetiology of recurrent haemoptysis in a child.

**Case presentation:**

A 4 years old girl presented with history of recurrent haemoptysis. Bronchoscopic evaluation excluded a foreign body aspiration but revealed right bronchial mucosal hyperaemia and varices. Diagnosis of right unilateral PVA was suspected on transthoracic echocardiography which demonstrated hypoplastic right pulmonary artery and non-visualization of right pulmonary veins. Final diagnosis was confirmed on cardiac CT angiography. A conservative treatment approach was opted with consideration for pneumonectomy in future when she is older.

**Conclusion:**

Rarer causes should be considered when investigating for recurrent haemoptysis in children. Bronchoscopy and cardiac imaging are useful tools to establish the diagnosis of unilateral PVA in our case.

## Bacground

Haemoptysis is an uncommon presenting symptom in children. The most common aetiologies are acute lower respiratory tract infection and foreign body aspiration [1, 2]. Diagnosis can be readily attained by history, physical examination and chest radiograph in most cases. With appropriate treatment, the clinical course is usually self-limiting. However, when the presentation is recurrent and initial assessment fails to establish the common causes, other rarer and potentially serious aetiologies should be explored.

We report a rare case of right unilateral pulmonary vein atresia as the underlying aetiology of recurrent haemoptysis in a child.

## Case presentation

**Fig. 1:**
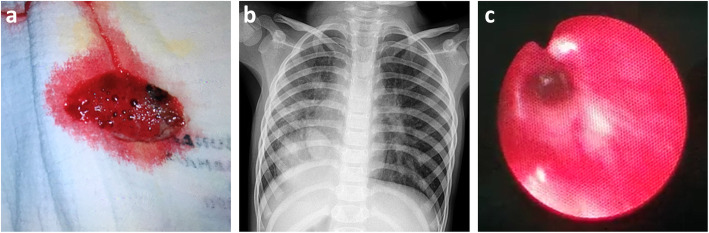
**a** Expectorant consisted of fresh bright blood mixed with small clots and mucus. **b** Chest radiograph showing ambiguous situs (abnormal liver and stomach position), mesocardia and a mass over the right para-cardiac region. **c** Bronchoscopic image of right main bronchus showing mucosal hyperaemia, oedema and variceal engorgement of submucosal blood vessels

A 4 years old girl was referred to our hospital for suspected foreign body aspiration. She presented with 3 days history of cough and haemoptysis. The expectorant consisted of fresh bright blood mixed with small clots and mucus (Fig. 1a). There were 2 previous similar episodes when she was 8 months and 3 years old. Both episodes were treated as Mallory-Weiss syndrome in another hospital and symptoms resolved without further investigation.

On physical examination, she was pink and not tachypnoeic. Her oxygen saturation was 98% under the room air. A grade 2 systolic murmur was detected over the right upper sternal border. There was reduced breath sound over the right lung field. Other organ system examination was unremarkable.

Chest radiograph (Fig. 1b) showed ambiguous situs (abnormal liver and stomach position), mesocardia and a mass over the right para-cardiac region. Both lung fields were clear. The complete blood count, coagulation profile and renal function were normal.

The initial chest CT performed at referring hospital revealed an intratracheal linear structure suspicious of foreign body.

An urgent flexible bronchoscopic evaluation was performed under general anaesthesia. No foreign body was detected but a prominent vertical mucosal fold was seen above the carina which explained the initial CT finding. The right main bronchus was significantly smaller in calibre compared to the left with mucosal hyperaemia, oedema and variceal engorgement of submucosal blood vessels (Fig. 1c).

Transthoracic echocardiogram was performed as part of the workout for heart murmur. It revealed left atrial isomerism and dextroposition of the heart. The right pulmonary artery was hypoplastic and the right pulmonary veins could not be traced. Otherwise, the rest of the intracardiac anatomy was normal and there was no evidence of pulmonary hypertension.

**Fig. 2:**
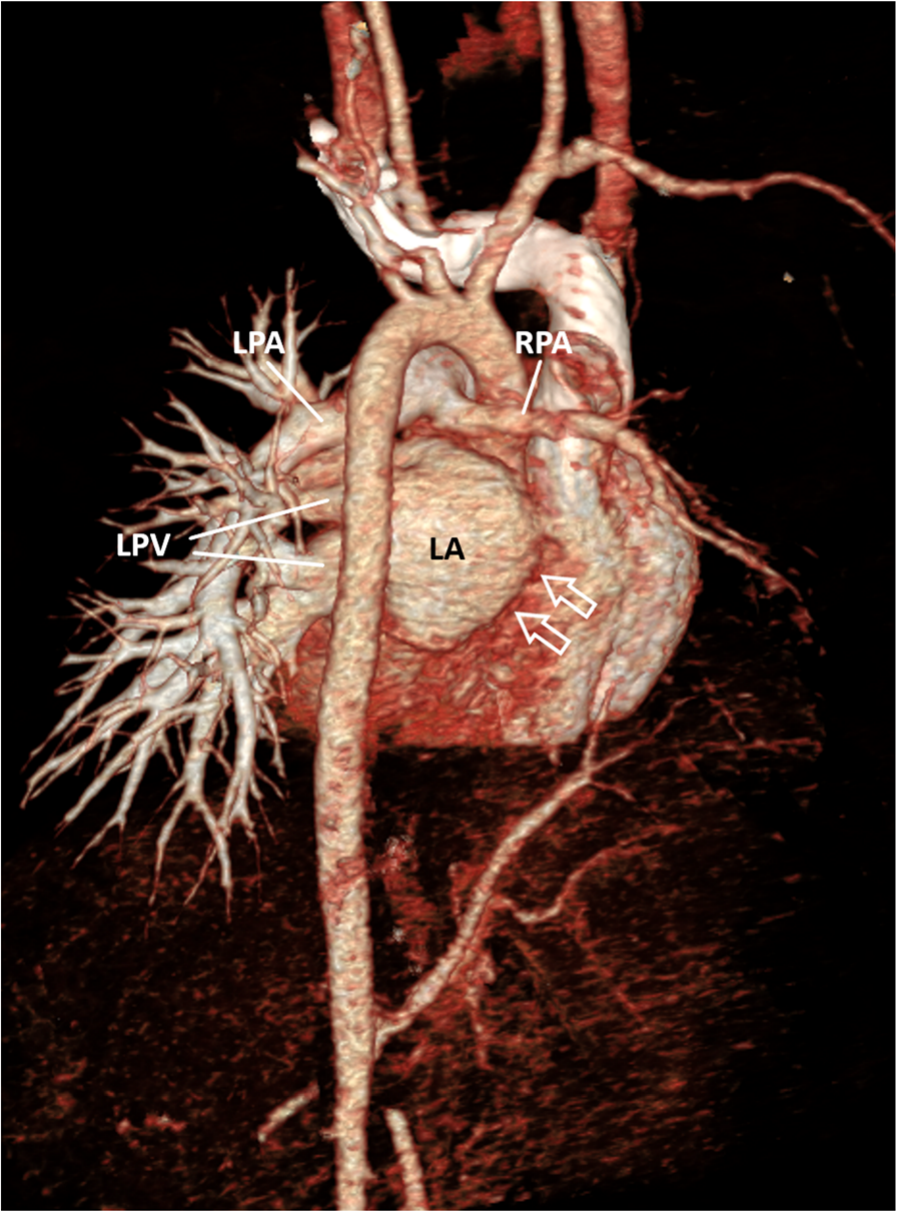
Volume-rendered reconstructed image of cardiac CT showing absence of right pulmonary veins with smooth appearance of adjacent posterior left atrial wall (white arrows). LA = left atrium, RPA = right pulmonary artery, LPA = left pulmonary artery, LPV = left pulmonary veins

**Fig. 3:**
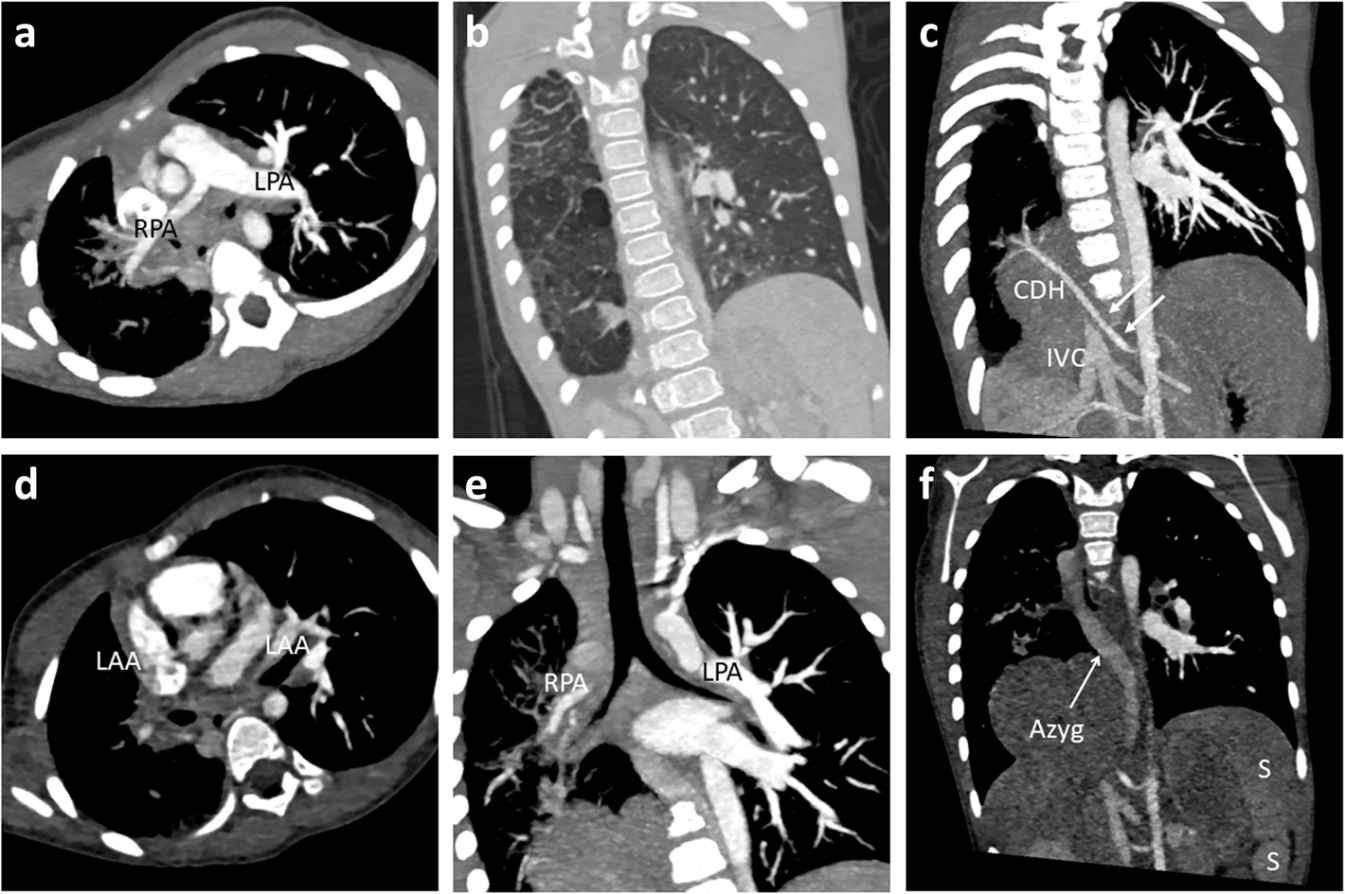
Multiplanar reconstructed cardiac CT images. **a** Significant size discrepancy between right and left pulmonary arteries. **b** Right lung parenchymal changes with diffuse interlobular septal thickening and mosaic attenuation. **c** Systemic-to-pulmonary collateral artery (white arrows) from the coeliac artery supplying the right lung. The hepatic segment of inferior vena cava is interrupted. **d** Both atrial appendages are long and narrow; characteristic of left atrial isomerism. **e** Both upper lobar bronchi course below the respective pulmonary arteries (hyparterial bronchi) which is typical of left bronchial isomerism. **f** Lobulated spleen which is a variant of polysplenia. Also shown is part of the dilated azygous vein. RPA = right pulmonary artery, LPA = left pulmonary artery, CHD = congenital diaphragmatic hernia, IVC = inferior vena cava, LAA = left atrial appendage, Azyg = azygous vein, S = spleen

Cardiac CT angiography confirmed the final diagnosis of right unilateral pulmonary vein atresia. The posterior left atrial wall at the expected site of right pulmonary veins entrance were completely smooth (Fig. 2). The right pulmonary artery was significantly smaller than the left with general paucity of right lung vasculature (Fig. 3a). The right lung volume was small with interlobular septal thickening and mosaic attenuation (Fig. 3b). In addition, there was a small systemic-to-pulmonary collateral artery from the coeliac artery supplying the lower lobe of the right lung (Fig. 3c). The CT also revealed a right diaphragmatic hernia containing part of the liver and complete viscerocardiac left isomerism (bilateral morphological left atrial appendages, bilateral hyparterial bronchi, interrupted inferior vena cava with azygous vein continuation and polysplenia) (Fig. 3d to f).

A multidisciplinary team meeting was held to discuss on the best treatment strategy. In view of self-limiting nature of each haemoptysis episodes, absence of serious complications such as recurrent severe respiratory infections or pulmonary hypertension and potential long-term sequelae of pneumonectomy in a young growing child, a conservative management approach with watchful follow up was adopted.

At 6 months after discharge, the patient remained symptom free.

## Discussion and conclusion

Haemoptysis is an uncommon but alarming presenting symptom in paediatric practice. The aetiologies differ significantly from that in adults. Haemoptysis in adults are commonly caused by tuberculosis, bronchogenic carcinoma and bronchiectasis but acute lower respiratory tract infection (tracheobronchitis, pneumonia) and foreign body aspiration constitute the two commonest causes of haemoptysis among children [1, 2]. Diagnostic workout frequently includes chest radiograph, chest CT and bronchoscopy [2, 3]. With appropriate treatment, the clinical course is usually self-limiting. However, other less common but serious aetiologies should be considered when the clinical course is recurrent or atypical and the initial assessment fails to establish the cause of the haemoptysis. These include congenital heart disease, cystic fibrosis, pulmonary arterial hypertension, Scimitar syndrome, airway neoplasms, vasculitis and pulmonary haemosiderosis [2].

In our case, echocardiogram provided important clues to the diagnosis by inability to detect presence of right pulmonary veins draining into the left atrium with corresponding ipsilateral pulmonary artery hypoplasia. Bronchial varices detected on bronchoscopy is a hallmark clinical feature of this disease [4] and final diagnosis was confirmed by cardiac CT angiography.

Unilateral pulmonary vein atresia is an extremely rare congenital malformation due to failure of incorporation of the common pulmonary vein into the left atrium [5]. Bilateral involvement is universally fatal at birth [6] but unilateral involvement allows longer survival; sometimes into adulthood [7]. Common clinical presentations are recurrent respiratory tract infections, dyspnoea and haemoptysis [8]. Haemoptysis is believed to be resulted from rupture of the dilated bronchial veins which form anastomosis with high-pressure obstructed pulmonary veins or systemic-to-pulmonary arterial collaterals. There is high association with other organ malformations and our case has concomitant left isomerism and right diaphragmatic hernia. Traditionally, confirmation of the diagnosis requires cardiac catheterization and invasive pulmonary artery angiography but characteristic findings on CT has allowed accurate diagnosis to be made non-invasively in most cases [9].

A possible differential diagnosis to be considered in our case is Scimitar syndrome which is also associated with right lung hypoplasia, dextroposition of the heart and systemic collateral arterial supply to part of the right lung. However, Scimitar vein, the anomalous pulmonary vein which commonly drains into the inferior vena cava in Scimitar syndrome could not be demonstrated on the CT in our case.

Without treatment, long term prognosis is usually guarded due to risk of massive pulmonary haemorrhage and development of pulmonary hypertension. Successful surgical repair has been reported in cases with well-developed pulmonary venous confluence to reconnect to the left atrium using sutureless pericardial marsupialization technique [10]. Pneumonectomy remains the only feasible option in most cases with unfavourable pulmonary venous anatomy or minimal residual lung function [8]. Decision for pneumonectomy should be carefully weighed against potential long-term post-pneumonectomy complications of scoliosis and chest wall deformity, especially in young growing children [11]. In milder cases, delaying the surgery to later age with periodic follow up to look out for clinical worsening is an appropriate alternative management strategy. Due to rarity of this disorder, long term outcome data remains scarce.

In conclusion, rarer aetiologies should be considered when investigating children presenting with recurrent haemoptysis. Presence of bronchial varices on bronchoscopy and failure to demonstrate the right pulmonary venous drainage with corresponding ipsilateral pulmonary artery hypoplasia on transthoracic echocardiogram have provided important clues leading to final diagnosis of unilateral pulmonary vein atresia by cardiac CT angiogram in our case.

## Data Availability

The datasets used and/or analysed during the current study are available from the corresponding author on reasonable request.

## Abbreviations

PVA: Pulmonary vein atresia
CT: Computed tomography
